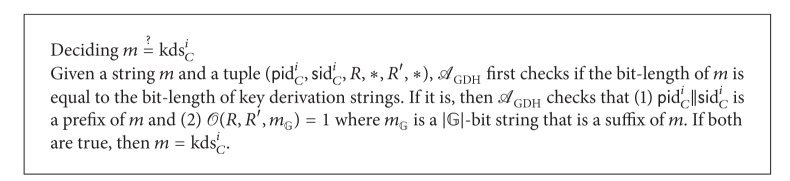# Password-Only Authenticated Three-Party Key Exchange Proven Secure against Insider Dictionary Attacks

**DOI:** 10.1155/2014/802359

**Published:** 2014-09-18

**Authors:** Junghyun Nam, Kim-Kwang Raymond Choo, Juryon Paik, Dongho Won

**Affiliations:** ^1^Department of Computer Engineering, Konkuk University, 268 Chungwondaero, Chungcheongbukdo, Chungju 380-701, Republic of Korea; ^2^Information Assurance Research Group, Advanced Computing Research Centre, University of South Australia, Mawson Lakes, SA 5095, Australia; ^3^Department of Computer Engineering, Sungkyunkwan University, 2066 Seoburo, Gyeonggido, Suwon 440-746, Republic of Korea

## Abstract

While a number of protocols for password-only authenticated key exchange (PAKE) in the 3-party setting have been proposed, it still remains a challenging task to prove the security of a 3-party PAKE protocol against insider dictionary attacks. To the best of our knowledge, there is no 3-party PAKE protocol that carries a formal proof, or even definition, of security against insider dictionary attacks. In this paper, we present the first 3-party PAKE protocol proven secure against both online and offline dictionary attacks as well as insider and outsider dictionary attacks. Our construct can be viewed as a protocol compiler that transforms any 2-party PAKE protocol into a 3-party PAKE protocol with 2 additional rounds of communication. We also present a simple and intuitive approach of formally modelling dictionary attacks in the password-only 3-party setting, which significantly reduces the complexity of proving the security of 3-party PAKE protocols against dictionary attacks. In addition, we investigate the security of the well-known 3-party PAKE protocol, called GPAKE, due to Abdalla et al. (2005, 2006), and demonstrate that the security of GPAKE against online dictionary attacks depends heavily on the composition of its two building blocks, namely a 2-party PAKE protocol and a 3-party key distribution protocol.

## 1. Introduction

Key exchange protocols (also known as key establishment protocols) enable two or more parties communicating over a public network to establish a shared secret key. This secret key, called a* session key*, is then used for building a confidential or integrity-preserving communication channel between the involved parties. Typically, a key exchange protocol is integrated with an authentication mechanism so that each party can ensure that the session key is in fact shared with the intended parties and not with an impostor. Achieving authenticated key exchange (AKE) inevitably requires some secret information to be preestablished between the parties during an initialization phase. Password-only authenticated key exchange (PAKE) protocols, for example, are designed to work when the preestablished secret information for authentication is only a human-memorable password.

The design of secure PAKE protocols continues to be a subject of active research. A major challenge in designing a PAKE protocol is to prevent dictionary attacks, in which an attacker exhaustively enumerates all possible passwords to find out the correct password. The difficulty of designing PAKE protocols secure against dictionary attacks is compounded in the 3-party setting, in which two clients wishing to establish a session key share their individual passwords only with an authentication server but not with any other client. A 3-party PAKE protocol must prevent potential dictionary attacks by a malicious client, who is registered with the server and thus is able to set up normal protocol sessions with other clients. (Throughout the paper, we will use the term “insider attacks” to refer to attacks mounted by a registered (malicious) client and use the term “outsider attacks” to refer to attacks mounted by a nonregistered party.) Indeed, a cursory review of the literature suggests that the majority of attacks mounted against 3-party PAKE protocols fall into the category of insider dictionary attacks; see, for example, Section 2 as well as [1–6].

The difficulties of obtaining a high level of assurance in the security of almost any new, or even existing, protocol are well illustrated with examples of errors found in many such protocols years after they were published. A widely accepted approach is to use a deductive reasoning process whereby the emphasis is placed on a proven reduction from the problem of breaking the protocol to another problem believed to be hard. Such an approach for key exchange protocols was made popular by Bellare and Rogaway [7] who provide the first formal definition for a model of adversary capabilities with an associated definition of the indistinguishability-based security. This model has been further revised several times, and one of the more recent revisions is the real-or-random (ROR) model proposed by Abdalla et al. [8, 9] for PAKE protocols. They also proposed a generic construction of a 3-party PAKE protocol that allows existing provably secure 2-party password-based key exchange and 3-party symmetric key distribution protocols to be* plugged and played* in a modular approach and yet remain secure [8, 9].

To date, a number of 3-party PAKE protocols have been presented with a formal proof of security [1, 8–17]. Most of these protocols were proven secure only in a restricted model, in which the adversary is not allowed to corrupt protocol participants [1, 8–10, 12–14]. In other words, these protocols were not proven secure against insider attacks including dictionary attacks conducted by insiders. Some protocols [11, 13] were subsequently found to be flawed [6, 18] and several protocols [15, 16] were proven secure only in a model that cannot capture (both insider and outsider) online dictionary attacks. Although protocols such as those of [19, 20] claimed to be provably secure against dictionary attacks of any kind, these protocols assume a “hybrid” 3-party setting where a server's public key is required in addition to passwords (see [21–27] for other protocols designed to work in a hybrid setting). To the best of our knowledge, no 3-party PAKE protocol has been proven secure against insider dictionary attacks.

We regard the contributions of this paper to be threefold.

*Contribution 1*. We present* the first 3-party PAKE protocol, a hashed variant of the protocol of  [1], whose indistinguishability-based security as well as password security against all classes of dictionary attacks are formally proved in a well-defined communication model*. Similar to the protocols of [1, 8, 9], our proposed protocol is generic in the sense that it can be constructed from any 2-party AKE protocol. Our construct can be viewed as a protocol compiler that transforms any 2-party AKE protocol into a 3-party AKE protocol with 2 more rounds of communication. If the given 2-party protocol is password-only authenticated, then the 3-party protocol output by the compiler will also be password-only authenticated. We prove the security of our construct in the random oracle model under the gap Diffie-Hellman (GDH) assumption; see Section 4.

*Contribution 2.* We offer* a simple and intuitive approach of capturing dictionary attacks* in the widely accepted model of Bellare et al. [28]. First, we clarify the relationship between the indistinguishability-based security of session keys and the password security against dictionary attacks.

- The indistinguishability-based security property in the Bellare-Pointcheval-Rogaway model implies security against (both insider and outsider) offline dictionary attacks. We demonstrate this by showing that a protocol cannot achieve the indistinguishability-based security if it is not secure against an offline dictionary attack (see Section 3.3).
- The indistinguishability-based security property does not imply security against undetectable online dictionary attacks (referred to as “UD online dictionary attacks” in the remainder of this paper). It is important to note that a protocol may be insecure against a UD online dictionary attack but it can still achieve the indistinguishability-based security (see Section 3.3).

This observation allows us to exclude offline dictionary attacks from our consideration once we have proved the indistinguishability-based security. We then introduce a new security definition to capture UD online dictionary attacks. We claim that our approach, when compared to those of [19, 20] (where a separate security definition is introduced to capture both UD online and offline dictionary attacks), provides a more intuitive and simpler way of proving security against dictionary attacks (see Section 3).

*Contribution 3*. We revisit the generic protocol GPAKE of Abdalla et al. [8, 9] to provide a detailed analysis of its security against UD online dictionary attacks. (We note that the work of Wang and Hu [1] has only provided a sketch of an insider UD online dictionary attack against GPAKE.) GPAKE is parameterized with a 2-party PAKE protocol 2PAKE and a 3-party key distribution protocol 3KD. We found that the security of GPAKE against both insider and outsider UD online dictionary attacks depends heavily on the security properties provided by 2PAKE and 3KD. For example, we can launch an insider UD online dictionary attack against the protocol GPAKE if neither 2PAKE nor 3KD provides client-to-server authentication. An outsider UD online dictionary attack can also be mounted under the same condition in addition to the condition that 2PAKE provides server-to-client authentication (see Section 2 for details).

## 2. Undetectable Online Dictionary Attacks against GPAKE

This section presents a comprehensive analysis of the security of the GPAKE protocol [8, 9] against UD online dictionary attacks. Our analysis shows that (1) GPAKE relies its security against UD online dictionary attacks on how its building blocks are instantiated and (2) the attacks could be mounted not only by a registered client (an insider) but also by a nonregistered party (an outsider). After conducting the security analysis, we suggest possible combinations of the instantiations that allow the GPAKE protocol to avoid the attacks.

### 2.1. A Review of GPAKE

The GPAKE protocol is constructed using a combination of three building blocks: (1) a 2-party PAKE protocol 2PAKE, (2) a 3-party key distribution protocol 3KD, and (3) a message authentication code- (MAC-) based Diffie-Hellman key exchange protocol MDH. Let *G* be a finite cyclic group generated by an element *g*; and let Σ = (*Mac*, *Ver*) be a MAC scheme where *Mac* and *Ver* are the MAC generation and MAC verification algorithms, respectively. The security of MDH relies on the hardness of the decisional Diffie-Hellman (DDH) problem in *G* and on the security of the underlying MAC scheme Σ against chosen message attacks. *S* represents a trusted server which registers clients and, during the registration, each client shares their individual password secretly with *S*. Suppose that *A* and *B* are two registered clients who wish to establish a session key and *pw*_*A*_ and *pw*_*B*_ are the passwords of *A* and *B*, respectively.

#### 2.1.1. Description

**Figure 1:**
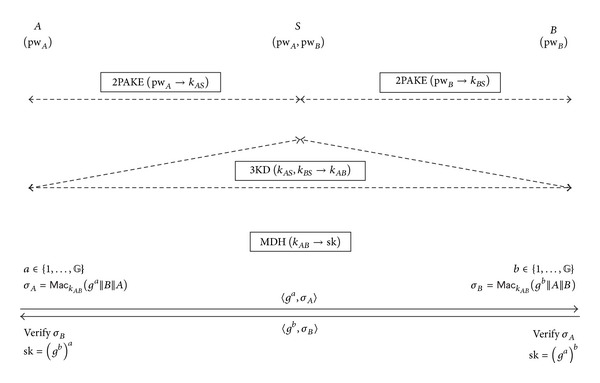
GPAKE: Abdalla et al.'s generic 3-party PAKE protocol [8, 9].

A high-level depiction of GPAKE is given in Figure 1 and its brief description follows. First, *A* (and *B*) and *S* establish a shared high-entropy key, *k*_*AS*_ (and *k*_*BS*_, resp.), by running the 2-party PAKE protocol, 2PAKE. Then, *S* generates a MAC key, *k*_*AB*_, and distributes *k*_*AB*_ to both *A* and *B* by running the 3-party key distribution protocol 3KD which takes *k*_*AS*_ and *k*_*BS*_ as input. A session key, sk = *g* ^*ab*^, will be established between *A* and *B* after running the MAC-based Diffie-Hellman key exchange protocol MDH which takes *k*_*AB*_ as an input.

#### 2.1.2. Instantiations

Abdalla et al. [8, 9] suggested several practical and provably secure protocols that can be used in the instantiation of the 2-party protocol 2PAKE. Among them are the KOY protocol [29, 30] and its generalization [31], the PAK suite [32], and other protocols based on encrypted key exchange (EKE) of Bellovin and Merritt [33] (e.g., Bresson et al.'s OMDHKE protocol [34]). For the instantiation of 3KD, any particular choice that is secure with respect to a single session will do since the symmetric keys given as input to 3KD differ from session to session. Bellare and Rogaway's 3PKD protocol [35], for example, was suggested as a possible choice. The MAC scheme used in MDH can be instantiated with the HMAC [36] and the CBC MAC.

### 2.2. An Insider Attack

**Figure 2:**
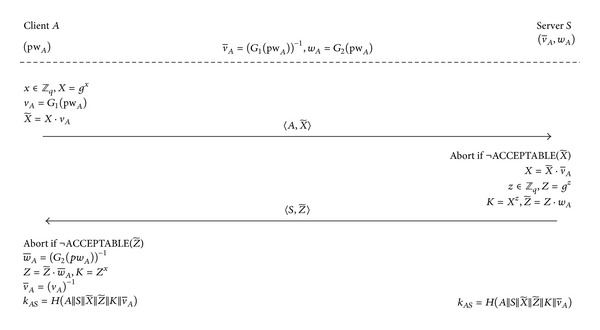
The PPK protocol [32].

**Figure 3:**
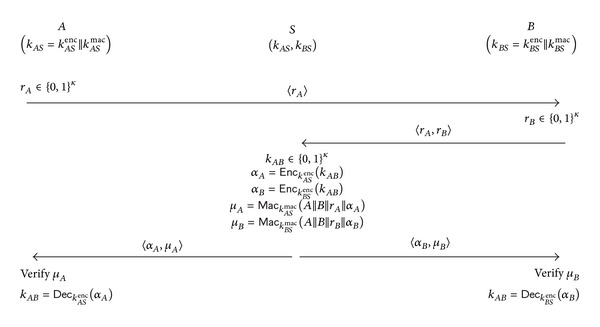
The 3PKD protocol [35].

Let us consider, for example, the case where 2PAKE and 3KD are instantiated with the PPK protocol [32] and with the 3PKD protocol [35], respectively. In this case, a malicious client *B* who is registered with the server, *S*, can mount a UD online dictionary attack against any other client, for example, *A*. It is important to note that we do not require either of the protocols, PPK (see Figure 2) and 3PKD (see Figure 3), to be insecure and, in fact, we assume that both protocols are secure.

#### 2.2.1. The PPK Protocol

Let *G* be a finite cyclic group of order *q*, and let *g* be a generator of *G*. The three hash functions used are *G*_1_ : {0,1}* → *G*, *G*_2_ : {0,1}* → *G*, and *H* : {0,1}* → {0,1}^*l*^, where *l* is the length of the session key. *pw*_*A*_ denotes *A*'s password known only to *A* and $\left({\overset{-}{v}}_{A},w_{A})=({\left(G_{1}(pw_{A})\right)}^{-1},G_{2}(pw_{A})\right)$ denotes the password verifiers held by *S*. The function acceptable  (∗) returns true, if and only if ∗ ∈ *G* (or, $*\in \hat{G}$ when *G* is defined as a (proper) subgroup of a group $\hat{G}$). The session key *k*_*AS*_ is computed as $k_{AS}=H(A||S||\tilde{X}||\tilde{Z}||K||{\overset{-}{v}}_{A})$. We observe that two messages $\langle A,\tilde{X}\rangle$ and $\langle S,\tilde{Z}\rangle$ are independent and thus the protocol can be easily modified to run in a single round.

#### 2.2.2. The 3PKD Protocol

The cryptographic tools used in 3PKD include (1) a symmetric encryption scheme consisting of a pair of encryption/decryption algorithms (*Enc*, *Dec*) and (2) a MAC scheme consisting of a pair of MAC generation/verification algorithms (*Mac*, *Ver*). The protocol runs between a trusted server, *S*, and two clients, *A* and *B*. *A* (and *B*) and *S* are assumed to have preestablished a 2*κ*-bit secret *k*_*AS*_ = *k*_*AS*_^enc^||*k*_*AS*_^mac^ (resp., *k*_*BS*_ = *k*_*BS*_^enc^||*k*_*BS*_^mac^). The protocol, depicted in Figure 3, begins by having *A* choosing a random *κ*-bit challenge *r*_*A*_ and sending it to client *B*. *B* also chooses a random *κ*-bit challenge *r*_*B*_ and sends 〈*r*_*A*_, *r*_*B*_〉 to *S*. Upon receiving 〈*r*_*A*_, *r*_*B*_〉, *S* generates a session key *k*_*AB*_ which he will distribute. *S* then encrypts *k*_*AB*_ under *A*'s encryption key *k*_*AS*_^enc^ (resp., *B*'s encryption key *k*_*BS*_^enc^) to get ciphertext *α*_*A*_ (resp., *α*_*B*_). Then, *S* computes *μ*_*A*_ (resp., *μ*_*B*_), the MAC under key *k*_*AS*_^mac^ (resp., *k*_*BS*_^mac^) of the string *A*||*B*||*r*_*A*_||*α*_*A*_ (resp. *A*||*B*||*r*_*B*_||*α*_*B*_). Then, *S* sends 〈*α*_*A*_, *μ*_*A*_〉 and 〈*α*_*B*_, *μ*_*B*_〉 to *A* and *B*, respectively. *A* and *B* accept the session key *k*_*AB*_ if and only if their received MAC is valid.

In order for PPK and 3PKD to be used together, the session keys generated by PPK need to be at least 2*κ*-bit long.

#### 2.2.3. One Possible Attack Scenario

The malicious client, *B*, can mount the following UD online dictionary attack against another client, *A*.

**Step 1 (running PPK).**

*B*  begins by initiating two concurrent runs, R1 & R2, of the PPK protocol.

- In the (dishonest) run R1, *B* impersonating *A* makes a guess (denoted by *pw*_*A*_′) on *A*'s actual password, *pw*_*A*_, selects a random *x* ∈ *Z*_*q*_, computes ${\tilde{X}}^{\prime }=g^{x}\cdot \;G_{1}(pw_{A}^{\prime })$, and sends the server *S* a fabricated message $\langle A,{\tilde{X}}^{\prime }\rangle$. Since ${\tilde{X}}^{\prime }\in G$, *S* will respond with the message $\langle S,\tilde{Z}\rangle$. After obtaining $\langle S,\tilde{Z}\rangle$, *B* computes a key $k_{AS}^{\prime }=H(A||S||{\tilde{X}}^{\prime }||\tilde{Z}||K^{\prime }||{\overset{-}{v}}_{A}^{\prime })$, where $K^{\prime }=(\tilde{Z}\cdot {\left(G_{2}{\left(pw_{A}^{\prime })\right)}^{-1}\right)}^{x}$ and ${\overset{-}{v}}_{A}^{\prime }={\left(G_{1}(pw_{A}^{\prime })\right)}^{-1}$. We let *k*_*AS*_′ = *k*′_*AS*_^enc^||*k*′_*AS*_^mac^.
- In the (honest) run R2, *B* honestly performs all the operations as per protocol specification and establishes the key, *k*_*BS*_ = *k*_*BS*_^enc^||*k*_*BS*_^mac^.

**Step 2 (running 3PKD).**

Once R1 & R2 are completed, *B* proceeds to run the protocol 3PKD by sending the server *S* two random challenges *r*_*A*_ and *r*_*B*_ (selected as specified by the protocol). *S* will respond to the random challenges with two messages 〈*α*_*A*_, *μ*_*A*_〉 and 〈*α*_*B*_, *μ*_*B*_〉. After obtaining these two messages, *B* recovers the keys  *k*_*AB*_′ = *Dec*_*k*′_*AS*_^ enc^_(*α*_*A*_) and  *k*_*AB*_ = *Dec*_*k*_*BS*_^ enc^_(*α*_*B*_).

**Step 3 (verifying the password guess).**

*B* verifies the correctness of *pw*_*A*_′ by checking that *k*_*AB*_′ is equal to *k*_*AB*_. If they are equal, *pw*_*A*_′ is the correct password with an overwhelming probability. Otherwise, it means that *pw*_*A*_′ ≠ *pw*_*A*_.

### 2.3. An Outsider Attack

**Figure 4:**
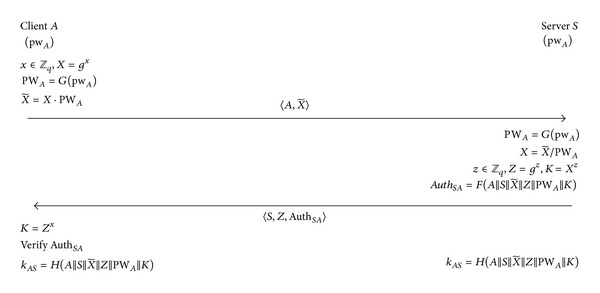
The OMDHKE protocol [34].

We now consider the case where 2PAKE and 3KD are instantiated with the OMDHKE protocol [34] and with the 3PKD protocol [35], respectively. Although OMDHKE (see Figure 4) is largely similar to PPK, there is a marked difference between the two. OMDHKE provides server-to-client authentication while PPK focuses on implicit key authentication.

#### 2.3.1. The OMDHKE Protocol

Let *G* be a finite cyclic group generated by an element *g* of prime order *q*. The hash functions used are *G* : {0,1}* → *G*, *H* : {0,1}* → {0,1}^*l*^, and *F* : {0,1}* → {0,1}^*f*^, where *l* is the length of the session key and *f* is the length of the authenticator Auth_*SA*_. Unlike the PPK protocol described in Figure 2, both *A* and *S* have a shared password, *pw*_*A*_. The protocol starts when *A* chooses a random *x* ∈ *Z*_*q*_, computes *X* = *g* ^*x*^, *PW*_*A*_ = *G*(*pw*_*A*_), and $\tilde{X}=X\cdot PW_{A}$, and sends $\langle A,\tilde{X}\rangle$ to *S*. Upon receiving the message from *A*, *S* computes *PW*_*A*_ = *G*(*pw*_*A*_) and $X=\tilde{X}/PW_{A}$, chooses a random *z* ∈ *Z*_*q*_, and computes *Z* = *g* ^*z*^, *K* = *X* ^*z*^, and $\text{Aut}{\text{h}}_{SA}=F(A||S||\tilde{X}||Z||PW_{A}||K)$. Then *S* sends 〈*S*, *Z*, Auth_*SA*_〉 to *A* and computes the session key, $k_{AS}=H(A||S||\tilde{X}||Z||PW_{A}||K)$. *A* computes *K* = *Z* ^*x*^, verifies Auth_*SA*_, and computes the session key $k_{AS}=H(A||S||\tilde{X}||Z||PW_{A}||K)$ if the verification succeeds.

Note that, in OMDHKE, server-to-client authentication is achieved via the authenticator Auth_*SA*_ sent by *S* to *A*.

#### 2.3.2. One Possible Attack Scenario

We now demonstrate how *C*, a malicious adversary who is not registered with the server, can mount a UD online dictionary attack against two registered clients, *A* and *B*.

**Step 4 (running OMDHKE).**

*C* initiates two concurrent runs, R1 & R2, of the protocol OMDHKE.

- In the (dishonest) run R1, *C* impersonating *A* makes a guess (denoted by *pw*_*A*_′) on *A*'s password, *pw*_*A*_, chooses a random *x* ∈ *Z*_*q*_, computes *PW*_*A*_′ = *G*(*pw*_*A*_′) and ${\tilde{X}}^{\prime }=g^{x}\cdot PW_{A}^{\prime }$, and sends the fabricated message $\langle A,{\tilde{X}}^{\prime }\rangle$ to *S*. *C* will then receive the response, 〈*S*, *Z*, Auth_*SA*_〉, from *S* as per protocol specification.
- In the (dishonest) run R2, *C* proceeds as per R1 but impersonating *B* (instead of *A*) and making a guess (denoted as *pw*_*B*_′) on *B*'s password, *pw*_*B*_. Let Auth_*SB*_ denote the authenticator received (in response) from  *S*.

**Step 5 (running 3PKD).**

After R1 & R2 are completed, *C* runs the 3PKD protocol with *S* while impersonating both *A* and *B*. (This step provides no useful information to *C* but is required for the attack to go undetected.)

**Step 6 (verifying the password guess).**

*C* can then verify the correctness of *pw*_*A*_′ by computing ${\text{Auth}}_{SA}^{\prime }=F(A||S||{\tilde{X}}^{\prime }||Z||PW_{A}^{\prime }||Z^{x})$ and then checking if Auth_*SA*_′ is equal to Auth_*SA*_. If they are equal, *pw*_*A*_′ is the correct password (with an overwhelming probability). Otherwise, *C* knows that *pw*_*A*_′ ≠ *pw*_*A*_. Similarly, the correctness of *pw*_*B*_′ can be verified using the received Auth_*SB*_.

### 2.4. Discussion

The insider attack described in Section 2.2 works because neither PPK nor 3PKD provides client-to-server authentication. Indeed, the same attack also works if we replace the PPK protocol with the OMDHKE protocol [34], the EKE2 protocol [28], or the SPAKE protocol [37]. In other words, the GPAKE protocol becomes vulnerable to the insider attack when both 2PAKE and 3KD are instantiated with a protocol that does not provide client-to-server authentication. The outsider attack described in Section 2.3 works under the same circumstance but additionally exploits the fact that the OMDHKE protocol provides server-to-client authentication.

Informally, both attacks can be prevented if one of the two protocols, 2PAKE or 3KD, is instantiated with a protocol that provides client-to-server authentication. We observe that a typical 3-party key distribution protocol is not expected to provide client-to-server authentication, and hence, we suggest that the countermeasure targets the instantiation of 2PAKE. While some might also suggest that a round-optimal protocol (i.e., a protocol that runs in a single round) should be used in the instantiation of 2PAKE to achieve better efficiency, we caution against this as no round-optimal 2-party PAKE protocol is known to provide client-to-server authentication and achieve security against offline dictionary attacks.

## 3. Modelling Dictionary Attacks in the Password-Only 3-Party Setting

The ROR model used for security analysis of GPAKE [8, 9] does not allow the adversary to access the *Corrupt* oracle and thus cannot capture any kind of insider attacks, in particular, (UD) online and offline dictionary attacks by a malicious insider. The security definition associated with the ROR model intends to capture indistinguishability of session keys and does not consider mounting an online dictionary attack against a protocol to be a violation of the security of the protocol (see Section 3.3). Consequently, none of the online dictionary attacks presented in Section 2 can be captured in the model.

We begin this section by presenting a communication model adapted from the Bellare-Pointcheval-Rogaway 2000 model [28] to support key exchange in the password-only 3-party setting. Our communication model allows the adversary to ask *Corrupt* queries and thereby captures insider attacks (as well as forward secrecy and unknown key share attacks). We then define a typical indistinguishability-based security of session keys, which we call the* SK security*. As we demonstrate in Section 3.3, the SK security implies security against offline dictionary attacks but does not imply security against UD online dictionary attacks. We then introduce a separate security definition to capture UD online dictionary attacks. Unlike the approach of [19, 20] where a separate security definition is introduced to capture both online and offline dictionary attacks, we only need to prove the protocol secure against UD online dictionary attacks once we have proved that it is SK-secure.

### 3.1. The Communication Model

#### 3.1.1. Participants and Long-Term Keys

We denote *S* by a trusted authentication server and *C* by the set of all clients registered with *S*. During registration, each client *C* ∈ *C* selects a password, *pw*_*C*_, from a dictionary, *D*, and shares *pw*_*C*_ with *S* via a secure channel. *pw*_*C*_ is used as the long-term secret key shared between *C* and *S*. Any two clients, *C*, *C*′ ∈ *C*, may run a 3-party PAKE protocol *P* with *S* at any point in time to establish a session key. Let *U* = *C* ∪ {*S*}. A user, *U* ∈ *U*, may participate in multiple protocol sessions running, either serially or concurrently, with the same or different participants. Thus, at any given time, there could be multiple instances of a single user. Π_*U*_^*i*^ denotes instance *i* of user *U*. We say that a client instance, Π_*C*_^*i*^,* accepts* when it computes its session key, sk_*C*_^*i*^, in an execution of the protocol.

#### 3.1.2. Partnering

We say, informally, that two instances are* partners* if they participate in a protocol execution and establish a (shared) session key. Formally, partnering between instances is defined in terms of the notions of session and partner identifiers (See [38] on the role and the possible construct of session and partner identifiers as a form of partnering mechanism that enables the right session key to be identified in concurrent protocol executions.) Session identifier (*sid*) is a unique identifier of a protocol session and is usually defined as a function of the messages transmitted in the session (although this may not be possible in a multiparty protocol where not all participants have the same view).  *sid*_*U*_^*i*^ denotes the *sid* of instance Π_*U*_^*i*^. A partner identifier (*pid*) is a sequence of identities of participants of a specific protocol session. Instances are given as input a *pid* before they can run the protocol.  *pid*_*U*_^*i*^ denotes the *pid* given to instance Π_*U*_^*i*^.  Note that  *pid*_*C*_^*i*^ = 〈*C*, *C*′, *S*〉, where *C*′ is another client with whom Π_*C*_^*i*^ believes it runs the protocol. We say that two instances, Π_*C*_^*i*^ and Π_*C*′_^*j*^, are partners if the following holds: (1) both Π_*C*_^*i*^ and Π_*C*′_^*j*^ have accepted, (2)  *sid*_*C*_^*i*^ = *sid*_*C*′_^*j*^, and (3)  *pid*_*C*_^*i*^ = *pid*_*C*′_^*j*^.

#### 3.1.3. Adversary Capabilities

The probabilistic polynomial-time (ppt) adversary *A* is in complete control of all communications between users, and its capabilities are modeled via a predefined set of oracle queries described below.

- *Execute*  (Π_*C*_^*i*^, Π_*C*′_^*j*^, Π_*S*_^*k*^): this query models passive attacks against the protocol. It prompts an execution of the protocol between the instances Π_*C*_^*i*^, Π_*C*′_^*j*^ and Π_*S*_^*k*^ and returns the transcript of the protocol execution to *A*.
- *Send*  (Π_*U*_^*i*^, *m*): this query sends message *m* to instance Π_*U*_^*i*^, modelling active attacks against the protocol. Upon receiving *m*, the instance Π_*U*_^*i*^ proceeds according to the protocol specification. The message output by Π_*U*_^*i*^, if any, is returned to *A*. A query of the form *Send* (Π_*C*_^*i*^, start: 〈*C*, *C*′, *S*〉) prompts Π_*C*_^*i*^ to initiate the protocol with *pid*_C_^i^ = 〈*C*, *C*′, *S*〉.
- *Reveal*  (Π_*C*_^*i*^): this query captures the notion of known key security (it is often reasonable to assume that the adversary will be able to obtain session keys from any session different from the one under attack). The instance Π_*C*_^*i*^, upon receiving the query and if it has accepted, returns the session key, sk_*C*_^*i*^, back to *A*.
- *Corrupt*  (*U*): this query returns the password *pw*_*U*_ of *U*. If *U* = *S* (i.e., the server is corrupted), all clients' passwords stored by the server are returned. This query captures not only the notion of forward secrecy but also unknown key share attacks and insider attacks.
- *Test*  (Π_*C*_^*i*^): this query is used to define the indistinguishability-based security of the protocol. If Π_*C*_^*i*^ has accepted, then, depending on a randomly chosen bit *b*, *A* is given either the real session key sk_*C*_^*i*^ (when *b* = 1) or a random key drawn from the session-key space (when *b* = 0). Following the ROR model [8, 9], we allow *A* to ask as many *Test* queries as it wishes. All *Test* queries are answered using the same value of the hidden bit *b*. Namely, the keys output by the *Test* oracle are either all real or all random. But, we require that, for each different set of partners, *A* should access the *Test* oracle only once.

We describe the number of queries asked by an adversary as the* query complexity* of the adversary. The query complexity is represented as an ordered sequence of five nonnegative integers, *Q* = 〈*q*_exec_, *q*_send_, *q*_reve_, *q*_corr_, *q*_test_〉, where *q*_exec_, *q*_send_, *q*_reve_, *q*_corr_, and *q*_test_ refer to the numbers of queries that the adversary asked, respectively, to the *Execute*, *Send*, *Reveal*, *Corrupt*, and *Test* oracles.

### 3.2. Session Key (SK) Security

We now proceed to define the basic security, called the SK security, of a 3-party PAKE protocol. The notion of* freshness* is a key element in defining the SK security. Intuitively, a fresh instance is one that holds a session key which should not be known to the adversary *A*, and an unfresh instance is one whose session key (or some information about the key) can be known by trivial means. A formal definition of freshness follows.

**Definition 11 .**

An instance Π_*C*_^*i*^ is fresh unless one of the following occurs: (1) *A* queries *Reveal*  (Π_*C*_^*i*^) or *Reveal*  (Π_*C*′_^*j*^), where Π_*C*′_^*j*^ is the partner of Π_*C*_^*i*^; or (2) *A* queries *Corrupt*  (*U*), for some  *U* ∈ *pid*_*C*_^*i*^, before Π_*C*_^*i*^ or its partner Π_*C*′_^*j*^ accepts.

The SK security of a 3-party PAKE protocol *P* is defined in the context of Box 1.

Let  *Succ*_0_ be the event that *A* succeeds in the experiment **E** **x** **p**_0_. Let  *Adv*_*P*_(*A*) denote the advantage of *A* in attacking protocol *P* and be defined as  *Adv*_*P*_(*A*) = 2 · *Pr*⁡_*P*,*A*_[*Succ*_0_] − 1.

**Definition 12 .**

A 3-party PAKE protocol *P* is* SK-secure* if, for any ppt adversary *A* asking at most *q*_send_ *Send* queries,  *Adv*_*P*_(*A*) is only negligibly larger than *c* · *q*_send_/|*D*|, where *c* is a very small constant (usually around 2 or 4) when compared with |*D*|.

To quantify the security of protocol *P* in terms of the amount of resources expended by adversaries, we let *Adv*_*P*_(*t*, *Q*) denote the maximum value of  *Adv*_*P*_(*A*) over all ppt adversaries *A* with time complexity at most *t* and query complexity at most *Q*.

### 3.3. Password Security

#### 3.3.1. Capturing Offline Dictionary Attacks

The SK security described in Definition 12 implies security against offline dictionary attacks. In other words, a 3-party PAKE protocol *P* is not SK-secure if it is not secure against an offline dictionary attack. To demonstrate this, suppose that the protocol *P* is not secure against an offline dictionary attack whereby an attacker *B* can derive the password of any registered client *A*. Then we can construct an adversary *A*_*off*⁡_ who breaks the SK security of protocol *P* as follows.

*Corruption*. If *B* is a malicious insider, *A*_*off*⁡_ queries *Corrupt*  (*B*) to obtain the password *pw*_*B*_. Otherwise, *A*_*off*⁡_ skips this step.

*Dictionary Attack*. Next, *A*_*off*⁡_ runs the protocol *P* exactly in the way that *B* conducts its offline dictionary attack against client *A*. Note that *A*_*off*⁡_ can perfectly simulate *B*'s attack by using the disclosed password *pw*_*B*_ and by asking oracle queries appropriately. At the end of this step, *A*_*off*⁡_ will obtain the password *pw*_*A*_ of client *A* as a result of the attack.

*Impersonation*. Now, *A*_*off*⁡_ initiates a new protocol session by querying *Send*  (Π_*C*_^*i*^, start: 〈*A*, *C*, *S*〉), where Π_*C*_^*i*^ is an unused instance of an uncorrupted client *C*. *A*_*off*⁡_ runs this session as per the protocol specification, but simulating by itself all the actions of *A* (by using *pw*_*A*_). At the end of the session, the instance Π_*C*_^*i*^ will accept with its session key sk_*C*_^*i*^.

*Test*. Clearly the instance Π_*C*_^*i*^ is fresh, since (1) no *Reveal* query has been made on Π_*C*_^*i*^ or its partner (which does not exist in this case) and (2) no *Corrupt* query has been made against any of *A*, *C*, and *S*. Thus, *A*_*off*⁡_ may ask the *Test*  (Π_*C*_^*i*^) query. Since *A*_*off*⁡_ can compute the same session key as sk_*C*_^*i*^, the probability that *A*_*off*⁡_ correctly guesses the bit *b* chosen by the *Test* oracle is 1 and so is the advantage of *A*_*off*⁡_ in attacking the protocol. Then, by Definition 12, the protocol *P* is not SK-secure since the number of *Send* queries asked by *A*_*off*⁡_ is much smaller (i.e., nonnegligibly smaller) than |*D* | /*c*.

#### 3.3.2. Capturing Undetectable Online Dictionary Attacks

Unfortunately, the SK security does not imply security against UD online dictionary attacks. In other words, a 3-party PAKE protocol that is not secure against a UD online dictionary attack may be rendered SK-secure. Let us assume a 3-party PAKE protocol *P* that is susceptible to a UD online dictionary attack (e.g., the GPAKE protocol in Section 2). Then, we can construct an adversary *A*_*on*⁡_ who attacks protocol *P* with advantage 1. The construction of *A*_*on*⁡_ is the same as that of *A*_*off*⁡_, except that to correctly determine the password *pw*_*A*_, *A*_*on*⁡_ may have to ask *Send* queries as many times as *d* · |*D*| for some integer *d* ≥ 1. Note that verifying the correctness of a password guess may require more than one *Send* query to be asked. Even if  *Adv*_*P*_(*A*_*on*⁡_) = 1, the protocol *P* is still rendered SK-secure by Definition 12, as the following holds for some *c* ≥ 1:

(1) AdvP(Aon⁡)≤cd|D||D|.

This result is not surprising since we call a protocol SK-secure if mounting an online dictionary attack by asking *Send* queries is the best an adversary can do. However, we want to be able to distinguish UD online dictionary attacks from detectable online dictionary attacks and ensure that the best an adversary can do is to mount a detectable online dictionary attack. The following new definitions together provide a simple and intuitive way of capturing security against UD online dictionary attacks.

**Definition 13 (an online dictionary attack).**

The *Send*  (Π_*S*_^*k*^, *m*) query models an* online dictionary attack* if both the following are true at the time of the termination of instance Π_*S*_^*k*^: (1) *m* was not output by a previous *Send* query asked to an instance of *C* by which Π_*S*_^*k*^ believes *m* was sent and (2) the adversary *A* queried neither *Corrupt*  (*S*) nor *Corrupt*  (*C*).

In Definition 13, the first condition implies that a straightforward delivery of a message between instances is not considered as an online dictionary attack while the second condition implies that when *C*′ is the (assumed) peer of client *C*, the adversary *A* can corrupt the peer client *C*′ to mount an (insider) online dictionary attack. Note that our definition of an online dictionary attack does not impose any restriction on asking  *Reveal* queries.

Let *Undet* be the event that, in experiment **E** **x** **p**_0_, a server instance terminates normally when an online dictionary attack was mounted against the instance. We say that the adversary *A* succeeds in mounting an UD online dictionary attack if the event *Undet* occurs. Formally, we define protocol's security against UD online dictionary attacks as follows:

**Definition 14 .**

A 3-party PAKE protocol *P* is secure against a UD online dictionary attack if, for any ppt adversary *A* asking at most *q*_send_ *Send* queries,  *Pr*⁡_*P*,*A*_[*Undet*] is only negligibly larger than *c* · *q*_send_/|*D*|, where *c* is as defined in Definition 12.

## 4. A Compiler for 3-Party PAKE Protocols

We now present a protocol compiler that transforms any 2-party PAKE protocol into a 3-party PAKE protocol. If the given 2-party protocol is SK-secure, then the 3-party protocol output by the compiler is not only SK-secure but also secure against both insider and outsider UD online dictionary attacks. (We stress again that the SK security implies resistance against both insider and outsider offline dictionary attacks.) This is the case regardless of whether the underlying 2-party protocol provides client-to-server authentication or not. Our transformation does not require the use of a 3-party key distribution protocol and always takes only two additional rounds of communication. Hence, applying the compiler to a round-optimal 2-party PAKE protocol immediately yields a 3-party PAKE protocol running in three communication rounds.

Our generic construction, which we call H3PAKE (“*H*” for “hashed”), is a variant of NGPAKE that is the generic construction of Wang and Hu [1]. The key difference between H3PAKE and NGPAKE is in the computation of the session key. NGPAKE defines the session key simply as the Diffie-Hellman key *g* ^*xy*^, whilst H3PAKE defines the session key as  *H*(*pid*||*sid*||*g* ^*xy*^) where *H* is a cryptographic hash function. The difference in how session key is computed, together with a minor modification in the specifications of the protocol messages, results in a significant improvement on the security of the constructions. More specifically, we are now able to prove that H3PAKE is secure against insider dictionary attacks, unlike NGPAKE where it is unclear whether it can be proven secure against insider dictionary attacks. Note that the security of NGPAKE was proved in the ROR model that does not allow the adversary to ask *Corrupt* queries; and as shown in Section 3.3, protocols proven secure in such a model cannot claim provable security against insider attacks of any kind.

### 4.1. Preliminaries

We begin with the cryptographic primitives on which the security of our construction relies.

*Gap Diffie-Hellman (GDH) Assumption.* Consider a finite cyclic group *G* of prime order *q* where the operation is denoted multiplicatively. Since the order of *G* is prime, all the elements of *G*, except 1, are generators of *G*. Let *g* be a random generator of *G*. The GDH problem in *G* is to solve the computational Diffie-Hellman (CDH) problem in *G* when given an oracle *O*(·, ·, ·) that solves the decisional Diffie-Hellman (DDH) problem in *G*. The DDH oracle *O*(·, ·, ·), on input a triple (*g* ^*a*^, *g* ^*b*^, *C*) for *a*, *b* ∈ *Z*_*q*_, outputs 1 if and only if *C* = *g* ^*ab*^. We define the advantage of a ppt algorithm *A* in solving the GDH problem in *G* as  *Adv*_*G*_^ GDH^(*A*) = *Pr*⁡[*A* ^*O*(·,·,·)^(*G*, *q*, *g*, *g* ^*x*^, *g* ^*y*^) = *g* ^*xy*^: *x*, *y*∈_*R*_ *Z*_*q*_]. We say that the GDH assumption holds in *G* if  *Adv*_*G*_^ GDH^(*A*) is negligible for all ppt algorithms *A*. We denote by  *Adv*_*G*_^ GDH^(*t*) the maximum value of  *Adv*_*G*_^ GDH^(*A*) over all algorithms *A* running in time at most *t*.

*Message Authentication Codes*. A message authentication code (MAC) scheme Σ is a triple of efficient algorithms (*Gen*, *Mac*, *Ver*) where (1) the key generation algorithm *Gen* takes as input a security parameter 1^*l*^ and outputs a key *k* chosen uniformly at random from {0,1}^*l*^; (2) the MAC generation algorithm *Mac*  takes as input a key *k* and a message *m* and outputs a MAC (also known as a tag) *σ*; and (3) the MAC verification algorithm *Ver* takes as input a key *k*, a message *m*, and a MAC *σ* and outputs 1 if *σ* is valid for *m* under *k* or outputs 0 if *σ* is invalid. Let  *Adv*_Σ_(*A*) be the advantage of an adversary *A* in violating the strong existential unforgeability of Σ  under adaptive chosen message attacks. More precisely,  *Adv*_Σ_(*A*) is the probability that an adversary *A*, who mounts an adaptive chosen message attack against Σ with oracle access to  *Mac*_*k*_(·) and  *Ver*_*k*_(·), outputs a message/tag pair (*m*, *σ*) such that (1) *Ver*_*k*_(*m*, *σ*) = 1 and (2) *σ* was not previously output by the oracle  *Mac*_*k*_(·) as a MAC on the message *m*. We say that the MAC scheme Σ is secure if  *Adv*_Σ_(*A*) is negligible for every ppt adversary *A*. We use  *Adv*_Σ_(*t*, *q*_ mac _, *q*_ ver _) to denote the maximum value of  *Adv*_Σ_(*A*) over all ppt adversaries *A* running in time at most *t* and asking at most *q*_ mac_ and *q*_ ver_ queries to  *Mac*_*k*_(·) and  *Ver*_*k*_(·), respectively.

*2-Party PAKE Protocols*. H3PAKE takes as input a 2-party PAKE protocol 2PAKE. We assume that the given 2-party protocol 2PAKE outputs session keys distributed in {0,1}^*l*^ and is SK-secure against an adversary who is given access to all the oracles: *Send*, *Execute*, *Reveal*, *Corrupt*, and *Test*. Let  *Adv*_2*PAKE*_(*A*) be the advantage of an adversary *A* in breaking the SK security of 2PAKE. We require that, for any ppt adversary *A* asking at most *q*_send_ *Send* queries,  *Adv*_2*PAKE*_(*A*) is only negligibly larger than *q*_send_/|*D*|.  *Adv*_2*PAKE*_(*t*, *Q*) denotes the maximum value of  *Adv*_2*PAKE*_(*A*) over all ppt adversaries *A* with time complexity at most *t* and query complexity at most *Q*.

Additionally, H3PAKE uses a cryptographic hash function *H* mapping {0,1}* to {0,1}^*κ*^, where *κ* is a security parameter representing the length of session keys. *H* is modelled as a random oracle in our proof of security for H3PAKE.

### 4.2. Description of H3PAKE

**Figure 5:**
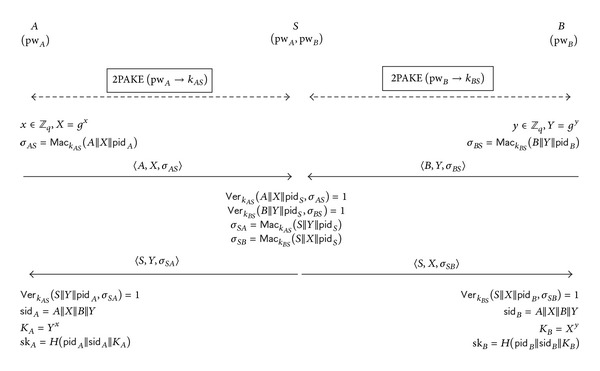
H3PAKE: our proposed generic 3-party PAKE protocol.

We assume that the following information has been preestablished and is known to all users in the network: (1) a cyclic group *G* of prime order *q* and a generator *g* of *G*, (2) a MAC scheme  Σ = (*Gen*, *Mac*, *Ver*), (3) a 2-party PAKE protocol 2PAKE, and (4) a cryptographic hash function *H*. These public parameters can be determined by the server and be broadcast to all its registered clients. Let *A* and *B* be two clients who wish to establish a session key, and let *S* be the trusted server with which *A* and *B* have registered their passwords *pw*_*A*_ and *pw*_*B*_, respectively. We denote the partner identifier *pid* given as input to (an instance of) *A* (resp., *B* and *S*) by  *pid*_*A*_ (resp.,  *pid*_*B*_  and  *pid*_*S*_). Recall that *pid* is a sequence of identities of protocol participants. The order of identities that appears in *pid* is of critical importance for the correctness of our construction and its security proof. For simplicity, we assume that  *pid*_*A*_ = *pid*_*B*_ = *pid*_*S*_ = 〈*A*, *B*, *S*〉.  Figure 5 depicts how the generic 3-party PAKE protocol, H3PAKE, is constructed from any given 2-party protocol, 2PAKE. More specifically, H3PAKE is constructed as follows.

*Ph* *as* *e 1*. *A* and *S* establish a shared high-entropy key *k*_*AS*_ by running the 2-party protocol 2PAKE. Likewise, *B* and *S* establish a shared high-entropy key *k*_*BS*_.

*Ph* *as* *e 2*. *A* and *B* establish their session key by running a MAC-based Diffie-Hellman key exchange protocol with assistance of *S*.

 Step 1. *A* chooses a random *x* ∈ *Z*_*q*_, computes *X* = *g* ^*x*^ and  *σ*_*AS*_ = *Mac*_*k*_*AS*__(*A*||*X*||*pid*_*A*_), and sends 〈*A*, *X*, *σ*_*AS*_〉 to *S*. Meanwhile, *B* chooses a random *y* ∈ *Z*_*q*_, computes *Y* = *g* ^*y*^ and *σ*_*BS*_ = *Mac*_*k*_*BS*__(*B*||*Y*||*pid*_*B*_), and sends 〈*B*, *Y*, *σ*_*BS*_〉 to *S*.
 Step 2. *S* checks that  *Ver*_*k*_*AS*__(*A*||*X*||*pid*_*S*_, *σ*_*AS*_) = 1 and   *Ver*_*k*_*BS*__(*B*||*Y*||*pid*_*S*_, *σ*_*BS*_) = 1. If either verification fails, *S* aborts the protocol. Otherwise, *S* computes  *σ*_*SA*_ = *Mac*_*k*_*AS*__(*S*||*Y*||*pid*_*S*_) and  *σ*_*SB*_ = *Mac*_*k*_*BS*__(*S*||*X*||*pid*_*S*_) and sends 〈*S*, *Y*, *σ*_*SA*_〉 and 〈*S*, *X*, *σ*_*SB*_〉 to *A* and *B*, respectively.
 Step 3. *A* verifies that  *Ver*_*k*_*AS*__(*S*||*Y*||*pid*_*A*_, *σ*_*SA*_) = 1. If the verification fails, *A* aborts the protocol. Otherwise, *A* sets the session identifier,  *sid*_*A*_ = *A*||*X*||*B*||*Y*, and computes the Diffie-Hellman key,  *K*_*A*_ = *Y* ^*x*^, and the session key,  *sk*_*A*_ = *H*(*pid*_*A*_||*sid*_*A*_||*K*_*A*_). Meanwhile, *B* checks if  *Ver*_*k*_*BS*__(*S*||*X*||*pid*_*B*_, *σ*_*SB*_) = 1 and aborts if the check fails. Otherwise, *B* sets  *sid*_*B*_ = *A*||*X*||*B*||*Y* and computes *K*_*B*_ = *X* ^*y*^ and  *sk*_*B*_ = *H* (*pid*_*B*_||*sid*_*B*_||*K*_*B*_).

At the end of the protocol execution, *A* and *B* will compute the same session key sk if they both hold the same sets of *pid* and *sid* and thus compute the same Diffie-Hellman key *K* = *g* ^*xy*^.

We do not require 2PAKE to be instantiated with a protocol that provides either unilateral or mutual authentication, as H3PAKE already provides mutual authentication between the server and the clients (via the MAC values exchanged in Phase 2). Hence, any 2-party protocol that provides implicit key authentication, including one-round protocols, will be suitable candidates to instantiate 2PAKE.

### 4.3. Proof of SK Security

We claim that the generic construction H3PAKE described in Figure 5 is SK-secure in the random oracle model under the GDH assumption in *G* and the security of the MAC scheme Σ.

**Theorem 15 .**

Let *H* be a random oracle. Then, for any adversary with time complexity at most *t* and query complexity at most *Q* = 〈*q*_*exec*_, *q*_*send*_, *q*_*reve*_, *q*_*corr*_, *q*_*test*_〉, its advantage in breaking the SK security of H3PAKE is bounded by

(2) AdvH3PAKE(t,Q)≤2·Adv2PAKE(t′,Q′)+2·qsend·AdvΣ(t′,2,2)+2·AdvGGDH(t′),

where *Q*′ = 〈2*q*_*exec*_, *q*_*send*_, *q*_*send*_, *q*_*corr*_, 2*q*_*exec*_ + *q*_*send*_〉 and *t*′ is the maximum time required to perform the experiment **E** **x** **p**_0_ involving an adversary who attacks H3PAKE with time complexity *t*.

*Proof.* Assume a ppt adversary *A* who attacks H3PAKE with time complexity *t* and query complexity *Q* = 〈*q*_exec_, *q*_send_, *q*_reve_, *q*_corr_, *q*_test_〉. We prove the theorem by making a series of modifications to the experiment **E** **x** **p**_0_, bounding the difference in *A*'s advantage between two consecutive experiments, and ending up with an experiment in which *A*'s advantage is negligible. By  *Succ*_*i*_, we denote the event that *A* correctly guesses the hidden bit *b* (chosen by the *Test* oracle) in experiment **E** **x** **p**_*i*_.

Before presenting the first modified experiment, we define the notion of a* clean* instance.

**Definition 16 .**

We say an instance Π_*U*_^*i*^ is unclean if *A* has queried *Corrupt*  (*U*′) for some *U*′ ∈ *pid*_*U*_^*i*^. Otherwise, we say it is clean.

*Experiment* **E** **x** **p**_1_.This experiment is different from **E** **x** **p**_0_ only in that we replace each different MAC key with a random key drawn uniformly from {0,1}^*l*^ for all clean instances. The difference in *A*'s advantage between **E** **x** **p**_0_ and **E** **x** **p**_1_ is bounded by the following lemma.

**Lemma 17 .**

|*Pr*⁡_*H*3*PAKE*,*A*_[*Succ*_1_] − *Pr*⁡_*H*3*PAKE*,*A*_[*Succ*_0_]| ≤ *Adv*_2PAKE_(*t*′, *Q*′), where *t*′ and *Q*′ are as defined in Theorem 15.

**Proof**

We prove the lemma by constructing an adversary *A*′ attacking protocol 2PAKE from the adversary *A* whose advantage in attacking H3PAKE is different between **E** **x** **p**_0_ and **E** **x** **p**_1_. *A*′ begins by choosing a bit *b* uniformly at random. Then, *A*′ runs *A* as a subroutine while simulating the oracles as follows.

*Ex* *ec* *ut* *e Queries*. When *A* makes an *Execute*  (Π_*A*_^*i*^, Π_*B*_^*j*^, Π_*S*_^*k*^) query, *A*′ first checks if any of *A*, *B*, and *S* was previously corrupted.

- If so, *A*′ answers the *Execute* query as in experiment **E** **x** **p**_0_.
- Otherwise, *A*′ answers the query using its own oracles. *A*′ first asks two queries *Execute*  (Π_*A*_^*i*^, Π_*S*_^*k*^) and *Execute*  (Π_*B*_^*j*^, Π_*S*_^*k*′^). Let  *T*_2*PAKE*_ and  *T*_2*PAKE*_′ be two transcripts returned in response to the *Execute* queries. Next, *A*′ makes the queries *Test*  (Π_*A*_^*i*^) and *Test*  (Π_*B*_^*j*^) and receives in return two keys ${\bar{k}}_{AS}$ and ${\bar{k}}_{BS}$ (either real or random). *A*′ then generates the rest of the protocol transcript (i.e., the messages to be sent in Phase 2), using ${\bar{k}}_{AS}$ and ${\bar{k}}_{BS}$ as the MAC keys. Finally, *A*′ returns these messages together with *T*_2*PAKE*_ and  *T*_2*PAKE*_′ after ordering them properly.

*Se* *nd* *Queries*. Whenever *A* makes a *Send*  (Π_*U*_^*i*^, *m*) query, *A*′ checks if *m* is a message for initiating a new session (of H3PAKE) or the *Send* query belongs to an execution of 2PAKE.

1. If both conditions are untrue, *A*′ responds to the query as in experiment **E** **x** **p**_0_.
2. Otherwise, *A*′ answers it by making the same query to its own *Send* oracle. If the query prompts Π_*U*_^*i*^ to accept, then *A*′ checks if anyone in  *pid*_*U*_^*i*^ was previously corrupted.
  - If so, *A*′ makes a *Reveal*  (Π_*U*_^*i*^) query and uses the output of this *Reveal* query as the MAC key of Π_*U*_^*i*^.
  - Otherwise, *A*′ makes a *Test*  (Π_*U*_^*i*^) query (unless the partner of Π_*U*_^*i*^ has already been tested) and uses the output of this *Test* query as the MAC key of Π_*U*_^*i*^.

*Re* *ve* *al* *Queries*. These queries are handled as in experiment **E** **x** **p**_0_.

*Co* *rr* *up* *t Queries*. *A*′ answers these queries in the straightforward way using its own *Corrupt* oracle.

*Te* *st* Queries. *A*′ answers these queries according to the bit *b* that it has chosen at the beginning of the simulation. That is, *A*′ returns real session keys, which it has computed on its own, if *b* = 1, and otherwise returns random keys chosen uniformly at random from {0,1}^*κ*^.

At some point in time, *A* will terminate and output its guess *b*′. When this happens, *A*′ outputs 1, if *b* = *b*′, and 0 otherwise.

From the simulation, it is obvious that

- the probability that *A*′ outputs 1 when its *Test*   oracle returns real session keys is equal to the probability that *A* correctly guesses the bit *b* in experiment **E** **x** **p**_0_;
- the probability that *A*′ outputs 1 when its *Test*  oracle returns random keys is equal to the probability that *A* correctly guesses the bit *b* in experiment **E** **x** **p**_1_.

This means that  *Adv*_2*PAKE*_(*A*′)  =  |*Pr*⁡_*H*3*PAKE*,*A*_[*Succ*_1_] − *Pr*⁡_*H*3*PAKE*,*A*_[*Succ*_0_]|.  Since *A*′ has at most time complexity *t*′ and query complexity *Q*′ = 〈2*q*_exec_, *q*_send_, *q*_send_, *q*_corr_, 2*q*_exec_ + *q*_send_〉, we obtain Lemma 17.

*Experiment* **E** **x** **p**_2_. Let *Forge* be the event that the adversary *A* makes a *Send* query of the form *Send*  (Π_*U*_^*i*^, *V*||∗||*σ*) before querying *Corrupt*  (*U*) and *Corrupt*  (*V*), where *σ* is a valid tag on  *V*||∗||*pid*_*U*_^*i*^ and was not output by a previous oracle query as a tag on  *V*||∗||*pid*_*U*_^*i*^. Then **E** **x** **p**_2_ is different from **E** **x** **p**_1_ only in that, if *Forge* occurs, the experiment is aborted and the adversary does not succeed. We claim the following lemma.

**Lemma 18 .**

|*Pr*⁡_*H*3*PAKE*,*A*_[*Succ*_2_] − *Pr*⁡_*H*3*PAKE*,*A*_[*Succ*_1_]| ≤ *q*_*send*_ · *Adv*_Σ_(*t*′, 2,2), where *t*′ is as defined in Theorem 15.

**Proof**

Given the adversary *A* attacking H3PAKE and assuming that the event *Forge* occurs, we construct an algorithm *F* that outputs, with a nonnegligible probability, a forgery against the MAC scheme Σ. The algorithm *F* is given oracle access to  *Mac*_*k*_(·) and  *Ver*_*k*_(·). The goal of *F* is to produce a message/tag pair (*m*, *σ*) such that (1)  *Ver*_*k*_(*m*, *σ*) = 1 and (2) *σ* was not previously output by the *Mac*_*k*_(·) oracle on input *m*.

Let *n* be the number of all different MAC keys that are established via a *Send* query of *A*. Clearly, *n* ≤ *q*_send_. *F* begins by choosing a random *α* ∈ {1,…, *n*}. Let *k*_*α*_ denote the *α*th key among all the *n* MAC keys, and let *Send*_*α*_ be a *Send* query that should be answered and/or verified using *k*_*α*_. *F* invokes *A* as a subroutine and handles the oracle calls of *A* as in experiment **E** **x** **p**_1_ except that it answers all  *Send*_*α*_ queries by accessing its MAC generation and verification oracles. As a result, the* α*th MAC key *k*_*α*_ is never used during the simulation. If *Forge* occurs against an instance that holds *k*_*α*_, *F* halts and outputs the message/tag pair generated by *A* as its forgery. Otherwise, *F* halts and outputs a failure indication.

If the guess *α* is correct, then the simulation is perfect and *F* achieves its goal. Namely,  *Adv*_Σ_(*F*) = *Pr*⁡[*Forge*]/*n*. Since *n* ≤ *q*_send_, we get  *Pr*⁡[*Forge*] ≤ *q*_send_ · *Adv*_Σ_(*F*). Then, Lemma 18 follows by noticing that *F* has at most time complexity *t*′ and makes at most two queries to  *Mac*_*k*_(·) and  *Ver*_*k*_(·).

*Experiment* **E** **x** **p**_3_. This experiment is different from experiment **E** **x** **p**_2_ only in that the *Execute* and *Send* oracles are simulated as in “the **E** **x** **p**_3_ modification” described in Box 2.

Since the view of *A* is identical between **E** **x** **p**_2_ and **E** **x** **p**_3_, following Lemma 19 is clear.

**Lemma 19 .**

*Pr*⁡_*H*3*PAKE*,*A*_[*Succ*_3_] = *Pr*⁡_*H*3*PAKE*,*A*_[*Succ*_2_].

In experiment **E** **x** **p**_3_, the advantage of *A* in attacking H3PAKE is bounded by the following lemma.

**Lemma 20 .**

*Pr*⁡_*H*3*PAKE*,*A*_[*Succ*_3_] ≤ (1/2) + *Adv*_*G*_^*GDH*^(*t*′) where *t*′ is as defined in Theorem 15.

**Proof**

The proof is via a reduction from the GDH problem which is believed to be hard. Assume that the advantage of *A* in attacking H3PAKE is nonnegligible. Then we can construct an algorithm *A*_GDH_ that has a nonnegligible advantage in solving the GDH problem in *G*. The goal of *A*_GDH_ is to compute and output the value *W*_3_ = *g* ^*w*_1_*w*_2_^ ∈ *G* when given a CDH-problem instance (*W*_1_ = *g* ^*w*_1_^, *W*_2_ = *g* ^*w*_2_^) ∈ *G* as well as an oracle *O*(·, ·, ·) that solves the DDH problem in *G*. *A*_GDH_ runs *A* as a subroutine while simulating all the oracles on its own.

When *A* asks an *Execute* and *Send* query, *A*_GDH_ answers it as specified in the **E** **x** **p**_3_ modification but using *W*_1_ and *W*_2_ instead of *V*_1_ and *V*_2_. In this way, *A*_GDH_ can embed the CDH-problem instance (*W*_1_, *W*_2_) into all protocol sessions. Accordingly, *A*_GDH_ can compute no session keys but can still correctly answer *Reveal* queries by storing all the keying materials associated with each instance. For each instance Π_*C*_^*i*^ whose only remaining work is to compute its session key, *A*_GDH_ checks if the instance Π_*C*_^*i*^ is clean or unclean. If it is clean, *A*_GDH_ stores a tuple  (*pid*_*C*_^*i*^,  *sid*_*C*_^*i*^,  *R*, *r*, *R*′, *r*′) into a list, which we denote as CDHList, where *R* = *W*_1_^*r*^ and *R*′ = *W*_2_^*r*′^. Here, the exponent *r* (resp., *r*′) is the one chosen for the instance whose user identity comes first (resp., second) in  *pid*_*C*_^*i*^. If it is unclean, *A*_GDH_ stores a tuple  (*pid*_*C*_^*i*^, *sid*_*C*_^*i*^, *R*, *r*, *R*′, ⊥) if *C* comes first in  *pid*_*C*_^*i*^ or a tuple  (*pid*_*C*_^*i*^, *sid*_*C*_^*i*^, *R*, ⊥, *R*′, *r*′) if *C* comes second in  *pid*_*C*_^*i*^. Here, ⊥ indicates that the exponent of the received public Diffie-Hellman value may have been chosen by *A*.

While imbedding the CDH-problem instance as above, *A*_GDH_ has to provide *A* with the same view as in experiment **E** **x** **p**_3_. To this end, let *kds* be a* key derivation string* from which a session key is computed by applying the random oracle *H*. Let *kds*_*C*_^*i*^ denote the *kds* of instance Π_*C*_^*i*^. Then,  *kds*_*C*_^*i*^ = *pid*_*C*_^*i*^||*sid*_*C*_^*i*^||*K*_*C*_^*i*^. As is clear from the above simulation, *A*_GDH_ cannot compute any *kds* on its own. But, given a string *m*, *A*_GDH_ can determine whether *m* is the *kds* of some instance Π_*C*_^*i*^ or not by repeatedly performing the deciding operation for the tuples in CDHList as in Box 3.

The simulation of other oracles is provided as follows.

* H Queries*. *A*_GDH_ uses a list, HList, to maintain input-output pairs of *H*. For each *H* query on a string *m*, *A*_GDH_ first checks if an entry of the form (*m*, *h*) is in HList. If it is, *A*_GDH_ returns *h* to *A*. Otherwise, *A*_GDH_ checks if *m* is the *kds* of some instance Π_*C*_^*i*^ by repeatedly performing the deciding operation above until a match is found.

- If a match is found and the corresponding tuple is of the form  (*pid*_*C*_^*i*^,  *sid*_*C*_^*i*^,  *R*, *r*, *R*′, *r*′), *A*_GDH_ computes *W*_3_ = (*m*_*G*_)^1/*rr*′^ and terminates outputting *W*_3_. In this case, *A*_GDH_ succeeds in solving the GDH problem.
- If a match is found and the corresponding tuple is of the form  (*pid*_*C*_^*i*^,  *sid*_*C*_^*i*^,   *R*, *r*, *R*′, ⊥) or  (*pid*_*C*_^*i*^,   *sid*_*C*_^*i*^,   *R*, ⊥, *R*′, *r*′), *A*_GDH_ checks if a tuple of the form  (*pid*_*C*_^*i*^,  *sid*_*C*_^*i*^,  *R*, *R*′, sk) is in the RList which is maintained by *A*_GDH_ to store revealed session keys. If it is, *A*_GDH_ returns sk to *A* and adds (*m*, sk) to HList. Otherwise, *A*_GDH_ returns a random *κ*-bit string *str* to *A* and adds (*m*, *str*) to HList.
- Otherwise, *A*_GDH_ returns a random *κ*-bit string *str* to *A* and adds (*m*, *str*) to HList.

*Reveal* *Queries*. When *A* asks a *Reveal*  (Π_*C*_^*i*^) query, *A*_GDH_ finds a tuple of the form  (*pid*_*C*_^*i*^,   *sid*_*C*_^*i*^, *R*, ∗, *R*′, ∗) in CDHList and checks if a tuple of the form  (*pid*_*C*_^*i*^,  *sid*_*C*_^*i*^, *R*, *R*′, sk) is in the RList. If it is, *A*_GDH_ returns sk to *A*. Otherwise, *A*_GDH_ checks if HList contains an entry (*m*, *h*) such that *m* = *kds*_*C*_^*i*^. Given the tuple  (*pid*_*C*_^*i*^,  *sid*_*C*_^*i*^,  *R*, ∗, *R*′, ∗), this check can be done by performing the deciding operation for all entries in HList. If such entry (*m*, *h*) exists in HList, *A*_GDH_ returns *h* in response to the query and adds the tuple (*pid*_*C*_^*i*^,  *sid*_*C*_^*i*^,  *R*, *R*′, *h*) into RList. Otherwise, *A*_GDH_ returns a random *κ*-bit string *str* to *A* and adds the tuple (*pid*_*C*_^*i*^,   *sid*_*C*_^*i*^, *R*, *R*′, *str*) into RList.

*Co* *rr* *up* *t Queries*. *A*_GDH_ answers these queries in the obvious way.

*Te* *s* *t Queries*. For each of these queries, *A*_GDH_ responses with a random *κ*-bit string.

Let *A* *sk* be the event that *A* makes an *H* query on a string *m* that is the *kds* of some fresh instance. From the simulation of *H*, it can be easily seen that as soon as *A* *sk* occurs, *A*_GDH_ outputs the desired result *W*_3_ = *g* ^*w*_1_*w*_2_^ and thus succeeds in solving the GDH problem in *G*. But, since *H* is a random oracle, *A* gains no advantage in distinguishing the test keys from random if the event *A* *sk* does not occur. This implies the assertion of Lemma 20.

This result combined with Lemmas 17–19 concludes the proof for Theorem 15.

### 4.4. Proof of Resistance to Undetectable Online Dictionary Attacks

We now claim that H3PAKE is secure against a UD online dictionary attack as long as the given 2-party protocol 2PAKE is SK-secure.

**Theorem 21 .**

Assume that, for any ppt adversary *A*′ asking at most *q*_*send*_ *Send* queries,  *Adv*_2*PAKE*_(*A*′) is only negligibly larger than *q*_*send*_/|*D*|. Then, for any ppt adversary *A* asking at most *q*_*send*_ *Send* queries,  *Pr*⁡_*H*3*PAKE*,*A*_[*Undet*] is only negligibly larger than *q*_*send*_/|*D*|, where *Undet* is as defined in Section 3.3.

**Proof**

Let *A* be an adversary who asks *q*_send_ *Send* queries in attacking the protocol H3PAKE. Assume that  *Pr*⁡_*H*3*PAKE*,*A*_[*Undet*] is nonnegligibly larger than *q*_send_/|*D*|. Given the adversary *A*, we prove the theorem by constructing an adversary *A*′ against 2PAKE who asks at most *q*_send_ *Send* queries but has an advantage nonnegligibly larger than *q*_send_/|*D*|.

*A*′ invokes *A* as a subroutine and answers the oracle queries of *A* as follows.

*Ex* *ec* *ut* *e Queries*. When *A* makes an *Execute*  (Π_*A*_^*i*^, Π_*B*_^*j*^, Π_*S*_^*k*^) query, *A*′ answers the query using its own *Execute* and *Reveal* oracles. *A*′ first queries *Execute*  (Π_*A*_^*i*^, Π_*S*_^*k*^) and *Execute*  (Π_*B*_^*j*^, Π_*S*_^*k*′^). Let  *T*_2*PAKE*_ and  *T*_2*PAKE*_′ be two transcripts returned in response to the *Execute* queries. Next, *A*′ obtains two keys *k*_*AS*_ and *k*_*BS*_ by querying *Reveal*  (Π_*A*_^*i*^) and *Reveal*  (Π_*B*_^*j*^). *A*′ then generates the rest of the protocol transcript, using *k*_*AS*_ and *k*_*BS*_ as the MAC keys. Finally, *A*′ returns these messages together with  *T*_2PAKE_ and  *T*_2*PAKE*_′ after ordering them properly.

*Se* *nd* *Queries.* When *A* makes a *Send*  (Π_*U*_^*i*^, *m*) query, *A*′ checks if *m* is a message for initiating a new session (of H3PAKE) or the *Send* query belongs to an execution of 2PAKE.

1. If both conditions are untrue, *A*′ responds to the query as that in the original experiment **E** **x** **p**_0_.
2. Otherwise, *A*′ answers it by making the same query to its own *Send* oracle. If the query prompts Π_*U*_^*i*^ to accept,  *A*′ checks if Π_*U*_^*i*^ is a server instance against which *A* has mounted an online dictionary attack. If not, *A*′ makes a *Reveal* (Π_*U*_^*i*^) query (and later uses the output of this *Reveal* query as the MAC key of Π_*U*_^*i*^). (How to handle the other case will be explained below.)

*Corrupt* *Queries*. *A*′ answers these queries using its own *Corrupt* oracle.

*Re* *ve* *al*/*Test* Querise. *A*′ answers these queries as in the original experiment **E** **x** **p**_0_.

Let Π_*S*_^*t*^ be any server instance against which *A* has mounted an online dictionary attack. Let  *k*_*S*_^*t*^ be the session key that the instance Π_*S*_^*t*^ has computed in its execution of 2PAKE. In order for the instance Π_*S*_^*t*^ to terminate normally, the adversary *A* has to make a query of the form *Send*  (Π_*S*_^*t*^, *C*||∗||*σ*_*CS*_) such that  *Ver*_*k*_*S*_^*t*^_(*C*||∗||*pid*_*S*_^*t*^, *σ*_*CS*_) = 1. When *A* makes such a *Send* query (i.e., when the event *Undet* occurs), *A*′ makes a *Test* query against the instance Π_*S*_^*t*^. Note that the instance Π_*S*_^*t*^ is fresh as (1) it is partnered with no instance and (2) *S* and *C* must have not been corrupted. Let ${\overset{-}{k}}_{S}^{t}$ be the key returned in response to the *Test* query. *A*′ outputs 1, if  ${Ver}_{{\overset{-}{k}}_{S}^{t}}(C||*||{pid}_{S}^{t},\sigma_{CS})=1$, and outputs 0, otherwise. If *Undet* does not occur, *A*′ outputs a random bit.

From the simulation above, it is clear to see that

(3) Adv2PAKE(A′) =2·Pr2PAKE,A′[Succ]−1 =2·(PrH3PAKE,A[Undet]+12(1−PrH3PAKE,A[Undet]))−1 =PrH3PAKE,A[Undet].

Then, Theorem 21 immediately follows since the number of *Send* queries asked by *A*′ against 2PAKE is at most *q*_send_.

## 5. Concluding Remarks

The undetectable online dictionary attacks we presented against the widely studied GPAKE protocol of Abdalla et al. [8, 9] are a reminder of the difficulty of designing a secure yet efficient 3-party PAKE protocol. The GPAKE protocol was proven secure in a model that does not capture undetectable online dictionary attacks, and thus, our attacks do not invalidate the proof of security for GPAKE.

We also presented a simple and intuitive approach of capturing all classes of dictionary attacks in the framework of the widely accepted Bellare-Pointcheval-Rogaway model. What motivated our approach is the observation that no prior work has provided a rigorous formal treatment of insider (online/offline) dictionary attacks in the password-only 3-party setting and as a consequence 3-party PAKE protocols insecure against such attacks have proliferated. We believe that our approach provides protocol designers with an easier and more accessible way of proving security of their protocols against dictionary attacks.

Finally, we presented a generic 3-party PAKE protocol (H3PAKE) and proved its security in the random oracle model under the gap Diffie-Hellman assumption. To the best of our knowledge, H3PAKE is the first 3-party PAKE protocol proven secure against both insider and outsider dictionary attacks as well as offline and online dictionary attacks. Future work includes coming up with a 3-party PAKE protocol that achieves the same (or even better) level of security and efficiency as H3PAKE but does not rely its security proof on the random oracle model.

## Figures and Tables

**Box 1:**
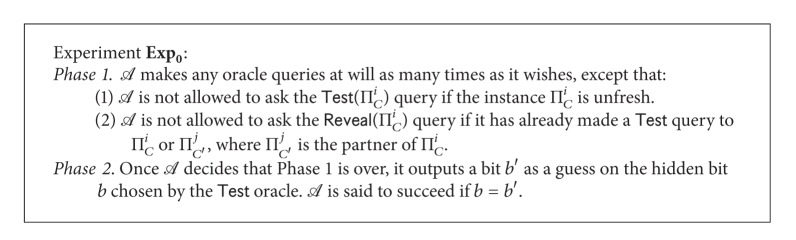


**Box 2:**
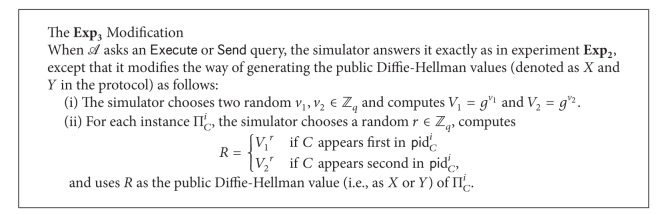


**Box 3:**